# Stoichiometric Shifts in Soil C:N:P Promote Bacterial Taxa Dominance, Maintain Biodiversity, and Deconstruct Community Assemblages

**DOI:** 10.3389/fmicb.2018.01401

**Published:** 2018-07-03

**Authors:** Zachary T. Aanderud, Sabrina Saurey, Becky A. Ball, Diana H. Wall, John E. Barrett, Mario E. Muscarella, Natasha A. Griffin, Ross A. Virginia, Albert Barberán, Byron J. Adams

**Affiliations:** ^1^Department of Plant and Wildlife Sciences, Brigham Young University, Provo, UT, United States; ^2^School of Mathematical and Natural Sciences, Arizona State University, Phoenix, AZ, United States; ^3^Department of Biology, School of Global Environmental Sustainability, Colorado State University, Fort Collins, CO, United States; ^4^Department of Biological Sciences, Virginia Polytechnic Institute, Blacksburg, VA, United States; ^5^Department of Plant Biology, University of Illinois Urbana-Champaign, Champaign, IL, United States; ^6^Environmental Studies Program, Dartmouth College, Hanover, NH, United States; ^7^Department of Soil, Water and Environmental Science, University of Arizona, Tucson, AZ, United States; ^8^Evolutionary Ecology Laboratories, and Monte L. Bean Museum, Department of Biology, Brigham Young University, Provo, UT, United States

**Keywords:** ecological stoichiometry, Lake Fryxell Basin, McMurdo Dry Valleys, network community modeling, nutrient colimitation, Solirubrobacteriaceae

## Abstract

Imbalances in C:N:P supply ratios may cause bacterial resource limitations and constrain biogeochemical processes, but the importance of shifts in soil stoichiometry are complicated by the nearly limitless interactions between an immensely rich species pool and a multiple chemical resource forms. To more clearly identify the impact of soil C:N:P on bacteria, we evaluated the cumulative effects of single and coupled long-term nutrient additions (i.e., C as mannitol, N as equal concentrations NH_4_^+^ and NO_3_^−^, and P as Na_3_PO_4_) and water on communities in an Antarctic polar desert, Taylor Valley. Untreated soils possessed relatively low bacterial diversity, simplified organic C sources due to the absence of plants, limited inorganic N, and excess soil P potentially attenuating links between C:N:P. After 6 years of adding resources, an alleviation of C and N colimitation allowed one rare Micrococcaceae, an *Arthrobacter* species, to dominate, comprising 47% of the total community abundance and elevating soil respiration by 136% relative to untreated soils. The addition of N alone reduced C:N ratios, elevated bacterial richness and diversity, and allowed rare taxa relying on ammonium and nitrite for metabolism to become more abundant [e.g., nitrite oxidizing *Nitrospira* species (Nitrosomonadaceae), denitrifiers utilizing nitrite (Gemmatimonadaceae) and members of Rhodobacteraceae with a high affinity for ammonium]. Based on community co-occurrence networks, lower C:P ratios in soils following P and CP additions created more diffuse and less connected communities by disrupting 73% of species interactions and selecting for taxa potentially exploiting abundant P. Unlike amended nutrients, water additions alone elicited no lasting impact on communities. Our results suggest that as soils become nutrient rich a wide array of outcomes are possible from species dominance and the deconstruction of species interconnectedness to the maintenance of biodiversity.

## Introduction

Environmental conditions dramatically structure soil bacterial communities; however, only a few environmental variables such as pH, salinity, and C substrate quality and quantity are known to drive community assemblages (Lozupone and Knight, 2007; Fierer et al., 2009; Baldrian et al., 2012). Borrowing from foundations in plant community ecology, following light and water, nutrient additions dramatically alter species abundance within communities often allowing certain species to dominate and biodiversity to decline (Bedford et al., 1999; Bobbink et al., 2010). For example, in a grassland, even low continual additions of N reduced species richness in just 2 years and depressed the number of species for as long as 20 years (Clark and Tilman, 2008). Classically, soil bacterial metabolism and growth is limited by water availability, then the quality and quantity of C substrates, and finally nutrient concentrations. But the role of major nutrients, such as N and P, remains incomplete even though changes in nutrient availability shape the responses of specific bacterial species or species interactions within soil communities (Marschner et al., 2003; Ramirez et al., 2010). Unlike plants, bacterial responses to resource constraints are complicated by interactions within consortia or communities where functionally disparate taxa (e.g., decomposers, nitrifiers, and methanogens) potentially dictate the form and availability of specific C substrates and nutrients necessary for other bacteria to become metabolically active and grow. In most soil communities such interactions are further complicated because soil organic C substrates are extremely numerous and diverse, containing both labile and more recalcitrant sources structuring the availability of N and P (Orwin et al., 2006; Hernandez and Hobbie, 2010). Furthermore, the C, N, and P requirements of bacterial biomass differ among species and ecosystems, and are not homeostatic through time (Cleveland and Liptzin, 2007; Hartman and Richardson, 2013).

Ecological stoichiometry is a unifying body of theory in ecology predicting relationships between the organismal biochemistry of plants, invertebrates, and microorganisms and the availability and recycling of nutrient elements in the environment (Elser and Hamilton, 2007). Ecological stoichiometry may also help identify the resource requirements of bacterial taxa and the conditions allowing certain bacteria to become metabolically active. Ecological stoichiometric theory was developed in aquatic ecosystems, but is universally valid, and over the last decades was also successfully applied to terrestrial systems (Redfield, 1958; Reiners, 1986; Cleveland and Liptzin, 2007; Austin and Vitousek, 2012). Soil (186:13:1) and soil microbial (60:7:1) C:N:P stoichiometry, like Redfield ratios for planktonic biomass (C:N:P = 106:16:1) (Redfield, 1958), are well-constrained across multiple biomes (Cleveland and Liptzin, 2007) offering incredible utility in understanding bacterial resource limitations and constraints on biogeochemical processes (Tian et al., 2010; Xu et al., 2013). The C:N:P stoichiometry of plant residues, soil organic matter, and bacterial biomass influence litter decomposition rates (Aneja et al., 2006; Zechmeister-Boltenstern et al., 2015), N and P mineralization rates (Mooshammer et al., 2012), and C-use-efficiency of bacteria, determining metabolic activity and trace gas flux (Keiblinger et al., 2010). Community structure is intimately connected to C:N:P ratios (Elser et al., 2000). Specifically, soil C:N:P stoichiometry sheds light on the potential for N and P availability to influence bacterial community structure. For example, higher N:P ratios in afforested soils of the Loess Plateau in China reflected P deficiencies among bacteria, leading to lower diversity but a higher abundance of Proteobacteria, Acidobacteria, and Nitrospirae (Ren et al., 2016). Further, a decrease in soil C:P ratios caused Gram-positive bacterial biomass to increase by 22% and the abundance of arbuscular mycorrhizal fungi to increase by 46% in a pasture following slash-and-burn agriculture in the South Ecuadorian Andes (Tischer et al., 2015). Taken together, investigating soil C:N, C:P, and N:P ratios are instrumental in identifying patterns of ecological coherence among responding bacteria under varying resource conditions.

Soil ecosystems of the McMurdo Dry Valleys, Eastern Antarctica are a model system for investigating stoichiometric controls over soil communities and ecosystem processes (Barrett et al., 2006). The extreme environment limits biota to cryptogrammic vegetation, a few taxa of metazoan invertebrates, and microbial dominated food webs. Phylum-level bacterial diversity in Antarctic soils is surprisingly high considering the environmental extremes and dearth of resources, i.e., organic matter and available nutrients (Cary et al., 2010; Lee et al., 2011). However, these soils host comparatively low diversity at the family or genus level relative to other biomes (Fierer et al., 2012), attenuating the nearly limitless possibilities of links between C:N:P stoichiometry and communities potentially present in high diversity ecosystems. Further, soil C:N:P ratios in most systems are necessarily complicated by plant residues with multiple different stoichiometric ratios that may mask links between C sources and release rate of N and P (Elser et al., 2000). Alternatively, due to the absence of vascular plants, Antarctic Dry Valley soils have some of the lowest soil organic matter concentrations on Earth (Burkins et al., 2000; Jobbagy and Jackson, 2000) with much of the soil organic matter being “legacy” C accumulated over thousands of years by cryptoendolithic bacteria, paleolake deposition, and minor inputs of contemporary algae and cyanobacteria from lakes, intermittent streams and saturated zones (Burkins et al., 2000). The concentrations of inorganic N and P are relatively low with N entering the system via atmospheric deposition (Michalski et al., 2005), endolithic and hypolithic cyanobacterial N_2_ fixation over millions of years (Cowan et al., 2014), and from dust, while P enters soils through mineral weathering (Barrett et al., 2007; Heindel et al., 2017). Both N and P concentrations vary among soils occurring on glacial tills with distinct exposure age and mineralogy (Barrett et al., 2007). Accompanying nutrient limitations, water is a universal resource essential for polar bacteria. In the McMurdo Dry Valleys, low precipitation inputs and sublimation and ablation processes (Fountain et al., 1999) cause dehydration stress and limit substrate diffusion to bacterial cells (Stark and Firestone, 1995). Water additions to Antarctic soils do create higher bacterial growth rates, elevate soil respiration, and decrease soil community diversity (Schwartz et al., 2014; Buelow et al., 2016). Therefore, water, in addition to nutrients, may have direct and indirect effects on community composition.

In this study, we explored the effects of long-term, coupled resource additions and water on bacterial species responses and ecosystem processes in a cold desert of Antarctica. After treating soils with six different resource additions including combinations of water, C as mannitol, N as equal NH_4_^+^ NO_3_^−^, and P as Na_3_PO_4_ annually over 6 years in the field, we evaluated shifts in bacterial community composition metrics such as richness, alpha diversity and evenness, taxa co-occurrence patterns, and soil respiration. Based on polar desert resource conditions, our initial soil C:N:P ratio (mean = 167:8:1, *n* = 8; more initial soil chemistry data is provided in the first section of the “Materials and Methods”), and the modal C:N:P ratio of soils (186:13:1, Cleveland and Liptzin, 2007), we hypothesized that C, N, CN, and CP additions will alleviate resource limitations and provide organic C and inorganic N for a subset of the community to exploit, while P is not limiting and adding more inorganic P will not alter community composition.

## Materials and Methods

### Study Site and Initial Soil Chemistry

Our study was conducted in a polar desert of the McMurdo Dry Valleys (76°30′ – 78°00′S, 160°00 – 165°00′E) at the McMurdo Long Term Ecological Research (LTER) site in Antarctica. The site was located in the Lake Fryxell basin (77°36.5′S, 163°14.9′E) of Taylor Valley, on Ross Sea drift soils (late-Quaternary) (Bockheim et al., 2008).

Dominant soil microflora include multiple species of algae from the division Chlorophyta and Heterokontophyta; microfauna comprised of nematodes, tardigrades and rotifers; and cyanobacteria, such species as, *Leptolyngbya frigida* and *Nostoc commune* in aquatic and terrestrial habitats (Adams et al., 2006). The basin experiences fewer than 50 days where average temperatures exceed 0°C within the summer months of December, January, February when mean annual temperature is -4.21°C ± 0.80 SD (*n* = 24). Soils receive less than 10 cm per yr^−1^ of effective mean annual precipitation falling as snow (Doran et al., 2002). Soils are Typic Haploturbels with shallow surface layer (≈0–10 cm depth), which experience continual cryoturbation, and a perennial permafrost layer (≈30–300 cm depth) (Bockheim and McLeod, 2008; Bockheim et al., 2008). All soils are poorly developed silty-loams with an average pH of 9.69 ± 0.12 SEM (*n* = 8) and an electrical conductivity of 258 ± 115 μS cm^−1^ (*n* = 8). Initial soil chemistry demonstrated that soils were generally extremely low in organic C (organic C = 0.03% ± 0.003, total soil C = 0.13% ± 0.01, *n* = 8) and possessed relatively high soil P (2.35 ± 0.18 μg g^−1^ soil, *n* = 8) but low levels of soil N of (0.003% ± 0.0004, *n* = 8).

### Stoichiometry and Water Long-Term Manipulations

To gain insights into the resource controls on microbial community assembly, we conducted a 6-year field stoichiometry experiment (austral summer field season 2006 – 2007 to 2011 – 2012) altering the stoichiometry of major nutrients (i.e., C, N, and P) and water availability. The experiment was a randomized block design with plots (1 m × 1 m) consisting of six treatments and an un-amended control: water only (W); C as mannitol and water (C); N as equal concentrations NH_4_^+^ and NO_3_^−^ with water (N); P as Na_3_PO_4_ and water (P); C, N, and water (CN); C, P, with water (CP); and the untreated control (U). The C additions, as mannitol, mirrored nutrient inputs from more contemporary algae and cyanobacteria. The N and P additions closely followed the Redfield ratio (106:16:1) to mimic new biomass from photosynthetic organisms entering organic matter-impoverished soils (Hecky et al., 1993). Annually, all nutrients were delivered as aqueous solutions to bring the soils to field capacity with concentrations of 15.3 g C m^−2^, 2.69 g N m^−2^ as NH_4_NO_3_, and 0.37 g P m^−2^ as Na_3_PO_4_12H_2_O and water of 12.7 L H_2_O m^−2^. For more information on the treatments and treatment application in the field see Ball et al. (2018). The present study comprises an analysis of the Fryxell basin site only.

### Soil C:N:P and Chemistry

To measure post-amendment changes in C:N:P stoichiometry we calculated C:N:P ratios from total C and N, and extractable P, and measured soil organic C and inorganic N. Soils were collected from all treatments (5 nutrient additions with water, 1 water addition, and a control × 8 replicates = 56) with a plastic scoop to a soil depth of 10 cm (approximately 500 g), sieved (2 mm sieve), and frozen until processing. Total C and N were measured on a Elantech Flash EA 1200 (CE Elantech, NJ, United States). Extractable soil P, as phosphate, was measured in 10 g soil with 0.5 M NaHCO_3_ (1:5 w/v) at pH 8.5, acidified with 3 mL of 6 N HCl, and analyzed on a Lachat Autoanalyzer (Barrett et al., 2007). We measured dissolved organic C on a Shimadzu TOC-5000A (Shimadzu Corporation, Columbia, MD, United States). Inorganic N (μg N-NH_4_^+^ g soil^−1^, μg N-NO_3_^−^ g soil^−1^) was evaluated from 20 g of soil extracted with 2 M KCl extraction (1:2.5 w/v), passed through a Whatman #1 filter, and measured on an a Lachat Quikchem 8500 (Lachat Instruments, Loveland, CO, United States). We tested for the effect of the additions on our response variables and soil C:N:P ratios using one-way ANOVA and Tukey’s HSD test to identify significant differences among the treatments in R (R Development Core Team, 2013). For stoichiometric analyses data were converted into molar ratios.

### Bacterial Community Responses to Changes in Soil C:N:P and Water

After maintaining the treatments for more than half a decade, we characterized soil communities in treatment soils using barcoded sequencing of the 16S rRNA gene. Soils were collected from three randomly selected replicates in all treatments (5 nutrient additions with water, 1 water addition, and a control × 3 replicates = 21) to a depth of 10 cm using sterile plastic scoops. All soils were transported from the field in an insulated chest, sieved to 2 mm, and stepped-down to −20°C over 24 h. Nucleic acids were extracted from 1.5 g of soil using a PowerSoil DNA Isolation Kit (Mo Bio Corporation, Carlsbad, CA, United States). We PCR amplified the V4 region of the16S rRNA gene using bacterial specific primer set 515F and 806R with unique 12-nt error correcting Golay barcodes (Caporaso et al., 2010; Aanderud et al., 2013). The thermal cycle conditions consisted of an initial denaturing step at 94°C for 3 min followed by 35 cycles of denaturing at 94°C for 45 s, annealing at 50°C for 30 s, and amplifying at 72°C for 90 s. After purifying (Agencourt AMPure XP PCR Purification Beckman Coulter Inc., Brea, CA, United States) and pooling PCR amplicons at approximately equimolar concentrations, samples were sequenced at the Brigham Young University DNA Sequencing Center^1^ using a 454 Life Sciences genome sequencer FLX (Roche, Branford, CT, United States). All sequences were trimmed and cleaned using mothur [v. 1.31.2; All sequences were trimmed and cleaned using mothur (v. 1.31.2^2^; Schloss et al., 2009)]. After removing barcodes and primers, we eliminated sequences that were <250 bp in length or possessed homopolymers longer than 8 bp. We then denoised the sequences with AmpliconNoise (Quince et al., 2011), removed chimeras using UCHIME (Edgar et al., 2011), and eliminated chloroplast, mitochondrial, archaeal, and eukaryotic 16S rRNA gene sequences based on reference sequences from the Ribosomal Database Project (Cole et al., 2009). We aligned the sequences against the SILVA database (Pruesse et al., 2007) with the SEED aligner, created operational taxonomic units (OTUs) based on uncorrected pairwise distances at 97% sequence similarity, and determined the phylogenetic identity of OTUs using the SILVA database.

To assess the effects of resources on soil bacterial communities, we first visualized differences in community composition using Principal Coordinates Analysis (PCoA) based on a Bray–Curtis distance matrix with the ‘vegan’ package in R (R Development Core Team, 2013). We then quantitated the effects of the different resource treatments [i.e., W, C, single nutrients N and P, and combined C and nutrients (CN and CP)] on community compositions using permutational multivariate analyses of variance (PERMANOVA) (Anderson, 2001) performed with the *adonis* function in the vegan package in R (Oksanen et al., 2013). Second, we quantified and constructed 95% confidence intervals for estimated richness as the total number of OTUs, alpha diversity as the inverse Shannon index, and taxa evenness using Pielous’s evenness based on 1000 iterations of 900 random resampled seqeunces from each replicate (Muscarella et al., 2014). Last, we calculated the relative recovery of nine phyla and three subclasses to identify differences in the distribution of major taxonomical groups (recovery ≥ 1.0%) due to the resource additions. Taxonomic trends of 24 families (recovery ≥ 1.0% in at least one replicate) were visualized in a heat map with hierarchal clustering using the *heatmap* function in the ‘gplot’ package in R (Warnes et al., 2014).

### Bacterial Community Network Models

To assess changes within communities at the OTU taxonomical level, we created network co-occurrence models for combinations of resources based on maximal information coefficient (MIC) analysis. We calculated all possible linear and non-linear associations between OTUs using the *mic* function and the ‘minerva’ package in R (Filosi et al., 2017), which belongs to a class of maximal information-based non-parametric exploration statistics for identifying and classifying relationships (Reshef et al., 2011). The nodes in the networks represented individual OTUs at 97% identity, while edges corresponded to valid or significant co-occurrence connections that occurred in at least 75% of all samples and had a MIC that was both >0.7 and statistically significant (*P-*value = 0.01) (Barberan et al., 2012). This filtering facilitated the determination of the OTUs interacting within the treatments and removed poorly represented OTUs reducing network complexity (Barberan et al., 2012). We described the network through a series of topological parameters: mean path length, mean degree, mean clustering coefficient, density, and modularity (Freedman et al., 2016). Network graphs in the *graphml* format were generated using ‘igraph’ package in R (Csardi and Nepusz, 2006) and visualized with the interactive platform Gephi (v. 0.8.2-beta) (Bastian et al., 2009). To identify the taxonomy of bacteria within the networks, we elevated nodes at the order taxonomical rank. We calculated the node number as the total number of nodes within each of the nineteen orders comprising the networks, and the relative recovery of nodes as the summation of the mean relative recoveries of the nodes within an a given order from P and CP or U and W communities.

### Soil Respiration

To investigate the links between microbial communities and ecosystem processes, we measured soil respiration (μmoles C-CO_2_ m^−2^ soil sec^−1^) in all resource treatments (5 nutrient additions with water, 1 water addition, and a control × 8 replicates = 56). Within 1 week of resource additions, soil CO_2_ flux in the field was evaluated using a Li-COR 8100 (LI-COR Biosciences, Lincoln, NE, United States) with a 10-cm diameter PVC ring inserted 2 cm into the soil at least 1 h prior to measurement (Ball et al., 2018).

## Results

### N and P Additions Altered Soil C:N:P

Resource additions clearly altered soil C:N:P leading to increases in N and P availability. Following multiple years of N additions, C:N were lower and N:P were higher in N and CN than P and CP soils (**Table 1**). The shifts in ratios were highlighted by inorganic NH_4_^+^ (one-way ANOVA, *df* = 6, *F* = 39.1, *P* < 0.001) and NO_3_^−^ (one-way ANOVA, *df* = 6, *F* = 14.9, *P* < 0.001) concentrations being at least forty-four- and nine-times higher, respectively, in N and CN than all other soil treatment (**Table 2**). Accompanying soil P additions, C:P were lower in P and CP soils than all other treatments besides CN (**Table 1**). Similarly, extractable P increased more than 53.0% in P and CP relative to W, C, and N (one-way ANOVA, *df* = 6, *F* = 6.26, *P* < 0.001, **Table 2**). Conversely, the additions of C had no apparent effect on total soil C only slightly increasing SOC in the C compared to P and W treatments (one-way ANOVA, *df* = 6, *F* = 3.80, *P* = 0.004, **Table 2**). The C:N:P ratios for each treatment are listed in **Table 1**.

**Table 1:** Molar C:N:P ratios of soils following 6 years of six resource additions in Fryxell lake basin of the Taylor Valleys in Antarctica.

	C:N:P	C:N	C:P	N:P
U	182:7:1	28.2 ± 2.69 ab	182 ± 29.7 ab	6.85 ± 1.01 bcd
W	247:8:1	31.6 ± 5.76 ab	247 ± 45.4 a	8.28 ± 0.768 bcd
C	218:8:1	30.0 ± 4.82 ab	218 ± 21.0 ab	8.43 ± 1.31 bc
N	223:16:1	14.4 ± 1.17 b	223 ± 30.8 a	15.5 ± 1.89 a
P	90:3:1	33.0 ± 49 a	90.3 ± 4.65 c	3.02 ± 0.400 d
CN	165:12:1	14.4 ± 1.68 b	165 ± 26.5 abc	11.9 ± 1.85 ab
CP	108:3:1	37.8 ± 6.00 a	108 ± 5.93 bc	3.47 ± 0.675 cd

*Treatments abbreviations include: un-amended control (U); water only (W); C as mannitol and water (C); N as equal concentrations NH_4_^+^ and NO_3_^−^ with water (N); P as Na_3_PO_4_ and water (P); C, N, and water (CN); C, P, with water (CP). Data are mean (±SEM, n = 8) with different letters indicating differences among treatments (P < 0.05) from ANOVA and Tukey’s HSD.*

**Table 2:** Soil chemistry following 6 years of resource additions.

	U	W	C	N	P	CN	CP
pH	9.7 ± 0.15	9.9 ± 0.05	9.9 ± 0.12	9.5 ± 0.20	9.8 ± 0.10	9.6 ± 0.17	9.8 ± 0.12
EC (ds m^−1^)	220 ± 42.1	183 ± 45.7	224 ± 40.9	331 ± 146	220 ± 42.0	301 ± 97.4	266 ± 73.8
Total C (%)	0.14 ± 0.01	0.12 ± 0.01	0.14 ± 0.01	0.14 ± 0.02	0.13 ± 0.01	0.15 ± 0.01	0.16 ± 0.02
Total N (%)	0.004 ± 0.001 bc	0.004 ± 0.001 c	0.005 ± 0.001 bc	0.008 ± 0.001 ab	0.004 ± 0.001 c	0.01 ± 0.001 a	0.004 ± 0.001 bc
P (μg N g soil^−1^)	3.6 ± 0.86 ab	2.2 ± 0.37 b	2.7 ± 0.39 b	2.5 ± 0.23 b	5.6 ± 0.67 a	4.2 ± 0.74 ab	5.8 ± 0.54 a
SOC (μg N g soil^−1^)	0.03 ± 0.004 ab	0.03 ± 0004 b	0.06 ± 0.008 a	0.03 ± 0.004 ab	0.03 ± 0.004 b	0.04 ± 0.007 ab	0.05 ± 0.005 ab
N-NH_4_^+^ (μg N g soil^−1^)	0.08 ± 0.09 b	0.27 ± 0.05 b	0.04 ± 0.01 b	17 ± 1.6 a	0.29 ± 0.10 b	13 ± 2.6 a	0.05 ± 0.01 b
N-NO_3_^−^ (μg N g soil^−1^)	0.64 ± 0.30 c	1.1 ± 0.57 c	0.09 ± 0.03 c	22 ± 4.2 a	0.77 ± 0.26 c	10 ± 2.5 b	1.1 ± 1.1 c

*For treatment abbreviations see **Table 1**. Data are mean (±SEM, n = 8) with different letters indicating significant differences among treatments (P < 0.05) based on ANOVA and Tukey’s HSD.*

### CN Reduced Evenness and Diversity but N Alone Enhanced Richness and Diversity

**FIGURE 1:**
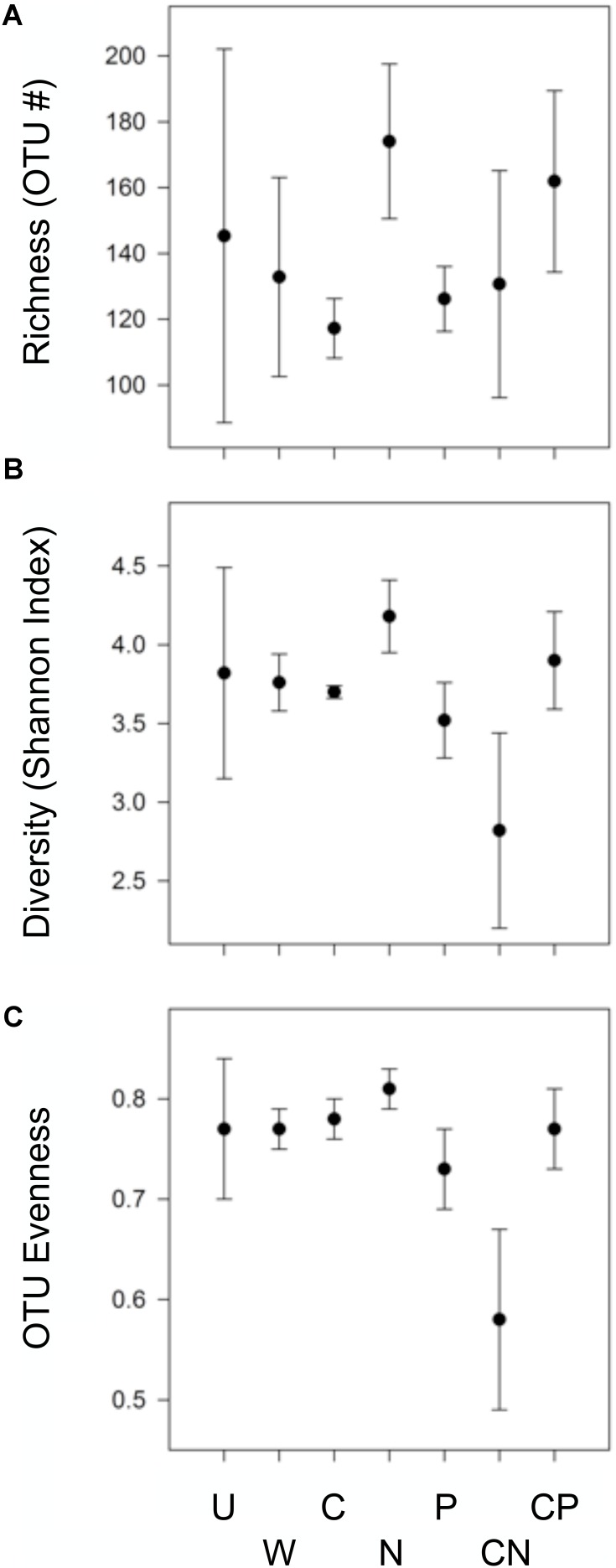
Only the addition of CN and N altered bacterial OTU richness and **(A)**, diversity **(B)**, or evenness **(C)**. Treatments include: carbon as mannitol (C), nitrogen as equal concentrations NH_4_^+^ and NO_3_^-^ (N), phosphorus (P), C and N (CN), C and P (CP), water only (W), and an un-amended control (UN). Values are means (*n* = 3) shown with accompanying 95% confidence intervals based on 16S rDNA community libraries (97% similarity cut-off).

Bacterial evenness and diversity was reduced following CN additions, while diversity and richness was enhanced by N additions (**Figure 1**). Specifically, the addition of CN dramatically depressed taxa evenness by at least 20.1% relative to all other treatments, and alpha diversity by at least 24.5% relative to the C, N, and CP treatments. In contrast, N additions stimulated OTU richness by 48.4 and 38.9% relative to bacterial communities in C and P soils, respectively. Diversity also increased by more than 13% in N compared to C, P, and CN treatments. In general, all resource additions reduced the variability surrounding richness and diversity metrics. All community inferences were based on the recovery of 138,458 quality sequences and 1,450 unique OTUs with samples possessing an average sequencing coverage of 98.4% ± 0.21 (mean and SEM). All sequence data were submitted to NCBI and are available as BioProject PRJNA476992.

### All Nutrient Additions Created Distinct Communities, Especially CN

**FIGURE 2:**
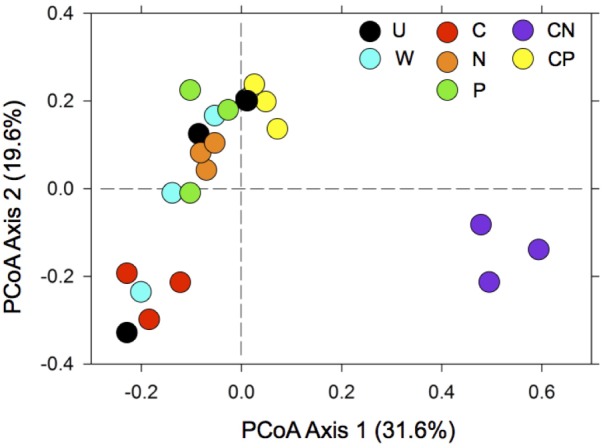
The addition of resources contributed to shifts in bacterial composition with the most dramatic change occurring in CN soils. For treatment abbreviations see **Table 1**. Treatments abbreviations include: C as mannitol (C), N as NH_4_^+^ and NO_3_^-^ (N), P as Na_3_PO_4_ (P), C and N (CN), C and P (CP), water only (W), and an un-amended control (U). The multivariate ordination was generated using principle coordinate analysis (PCoA) on a sample × OTU matrix of 16S rDNA community libraries (97% similarity cut-off).

The CN treatment dramatically influenced bacterial communities, most notably by the separation of communities along axis one, which explained 31.6% of the variation among communities (**Figure 2**). Further, the addition of any nutrient (i.e., C, N, P, and CP) reduced the variability among communities compared to the untreated control and water only addition along axis 2, which explained 19.6% of the variation. PERMANOVA results supported these interpretations, as communities were distinct among treatments (*F* = 3.5, *R*^2^ = 0.60, *P* < 0.001, *df* = 6).

### Family-Specific Responses to Nutrients

**FIGURE 3:**
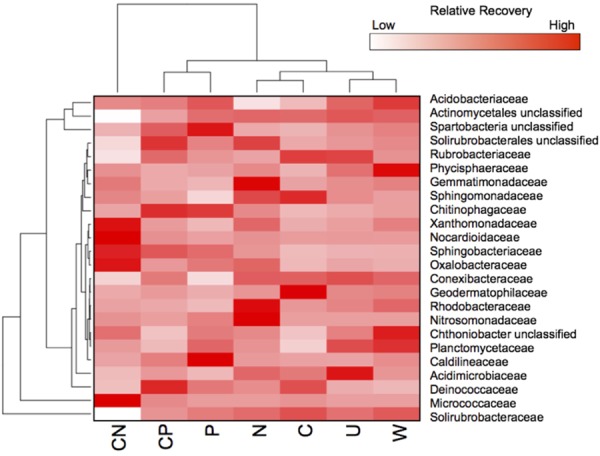
All resources, except water, promoted different taxonomical shifts. Heat map showing the distribution of OTUs for fourteen families that contributed ≥0.5% to the total recovery of communities. Treatment abbreviations are described in **Figure 1**. Values are based on means with hierarchal clustering of resource treatments (bottom) and family (left).

All resource additions, except water, promoted taxonomic shifts in bacterial OTU abundance in 11 families across five phyla (**Figure 3**). The most pronounced increase in relative recovery occurred in the Micrococcaceae (Actinobacteria) in CN soils where a single bacterium, an *Arthrobacter species*, was relatively rare (0.06% ± 0.05) in untreated soils but constituted 47% ± 5.6 of the community in CN-amended soils. The only other bloom occurred in the Trueperaceae (*Deinococcus)*, where one OTU was classified as intermediate (0.42% ± 0.09) in untreated but became abundant (9.6% ± 3.8) in CP soils. In general, Actinobacteria were abundant in all soils and accounted for at least 36% of the community composition in all treatments (Supplementary Figure S1), but not all Actinobacteria responded positively to CN. For example, three Actinobacteria families, Solirubrobacteracea, Solirubrobacterales unclassified, and Rubrobacteriaceae, decreased from 2.1- to 4.9-fold in CN compared to all other treatments (**Figure 3**). CN additions also stimulated Xanthomonadaceae (2.7% ± 1.2, Gammaproteobacteria) and Sphingobacteriaceae (1.8% ± 0.48, Bacteroidetes). With the addition of N, the Nitrosomonadaceae (Betaproteobacteria) increased in recovery 5.2-times allowing the N treatment to have the highest recovery of Betaproteobacteria (3.1% ± 0.17). Annual N additions also enhanced the recovery of Rhodobacteraceae (0.51% ± 0.16, Alphaproteobacteria) and Gemmatimonadaceae (2.7% ± 0.27, Gemmatimonadetes) by at least 1.8-times relative to all other treatments. Both CP and P additions enhanced the recovery of Chitinophagaceae (Bacteroidetes) and Spartobacteria unclassified (Verrucomicrobia) in particular. The recovery of these families was at least 1.5-times higher in P and CP than all other soils and caused the recovery of Bacteroidetes to increase upward of 100% (*P* = 6.3% ± 2.2, CP = 6.8% 1.8) in both treatments.

Deinococcus-Thermus were present in all soils (Supplementary Figure S1), but the addition of CP, C, and P stimulated the recovery of these taxa causing the Deinococcaceae to range from 5.3 to 11% (**Figure 3**). The addition of C alone promoted different families with Sphingomonadaceae (Alphaproteobacteria) and the Geodermatophilaceae (Actinobacteria) increasing upward of 3.0- and 39-times, respectively, but combined these two taxa only accounted for less than 4.1% of the community in C-amended soils.

### Network Community Modeling

**FIGURE 4:**
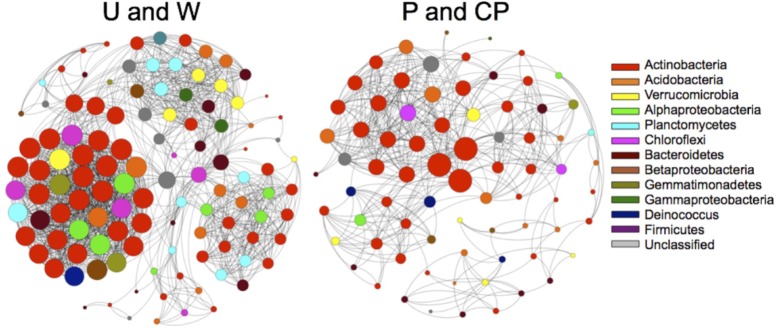
Community interactions among bacterial species deteriorated with the addition of P and CP. The two network models are based on OTUs from two 16S rDNA community libraries of Control and W, and P and CP treatments.

Nutrient additions disrupted interactions among community assemblages. Due to the requirement of more than three samples to create reliable community network models with MIC, we only created community network models for two combined treatments (U and W, and P and CP) that were relatively similar (PERMANOVA: U and W, *F* = 5.8, *R*^2^ = 0.59, *P* = 0.1, *df* = 1; PW and CPW, *F* = 1.2, *R*^2^ = 0.22, *P* = 0.5, *df* = 1). After years of P and CP additions, multiple aspects of the community broke down relative to the untreated and water only soils (**Figure 4** and **Table 3**). For example, the number of significant nodes or taxa, and edges or connections between taxa was 51 and 73% lower, respectively, in P and CP networks. The mean degree (number of connections per node to other nodes) declined twofold from U and W to P and CP networks, and mean path length (number of nodes needed to link any one node to any other in the network) decreased from 3.5 in the U and W to 2.8 in P and CP models. Within the two networks, specific orders were favored and the nodes were often major contributors to the recovery of the community. For example, the Trueperaceae represented 3 nodes in the combined P and CP network and 7.7% relative recovery in the P and the CP communities, but only 1 taxon in U and W network and 0.61% of the recovery in U and W communities. Alternatively, in the U and W network, Phycisphaerae (unclassified; 10 nodes, 2.1% relative recovery), Intrasporangiaceae (2 nodes, 0.13% relative recovery), and Xanthomonadaceae (2 nodes, 0.55% relative recovery) were present but completely absent from the P and CP network. The Spartobacteria (unclassified), Micrococcaceae, and Chitinophagaceae had similar numbers of nodes in all models, but contributed substantially more in abundance in the P and CP communities, 14, 3.2, and 3.1%, respectively. The Solirubrobacteriaceae and Solirubrobacterales (unclassified) consistently contributed to both models with 14 and 10 nodes in the P and CP, and the U and W networks, respectively, and comprised no less than 20% of the relative recovery from either community.

**Table 3:** Community network model characteristics for soil bacteria in P-amended and control soils.

Network topographical parameters	U and W		P and CP	
Nodes, edges (#)	113, 1321		75, 433	
Mean path length	2.8		3.5	
Mean degree	23		11.5	
Mean clustering coefficient	0.84		0.74	
Density	0.21		0.16	
Modularity	0.54		0.51	

	**U and W**		**P and CP**	
**Network taxonomy (order)**	**Node #**	**Relative recovery of nodes (%)**	**Node #**	**Relative recovery of nodes (%)**

Acidobacteriaceae	10	14	7	13
Intrasporangiaceae	2	0.13	0	0
Micrococcaceae	1	0.06	1	3.2
Nocardioidaceae	1	0.10	0	0
Rubrobacteriaceae	4	1.0	3	0.83
Conexibacteraceae	1	0.40	1	0.16
Solirubrobacteriaceae	2	1.3	4	2.0
Solirubrobacterales Unclassified	8	22	10	18
Chitinophagaceae	5	0.32	4	3.1
Sphingobacteriaceae	0	0	1	0.90
Caldilineaceae	1	0.28	1	1.5
Trueperaceae	1	0.51	3	7.7
Gemmatimonadaceae	1	0.65	1	0.16
Phycisphaerae unclassified	10	2.1	0	0
Sphingomonadaceae	1	0.98	2	1.8
Alcaligenaceae	1	0.12	1	0.14
Oxalobacteraceae	1	0.18	1	0.50
Xanthomonadaceae	2	0.55	0	0
Spartobacteria unclassified	4	7.3	4	14

*For treatment abbreviations see **Table 1** and for an explanation of network topographical parameters see network community modeling in the results section. For network taxonomy, node # is the total number of nodes or OTUs in a given order for a network. The relative recovery of nodes is the summation of mean relative recoveries of the nodes within the order from P and CP or U and W communities.*

### CN Elevated Soil Respiration

**FIGURE 5:**
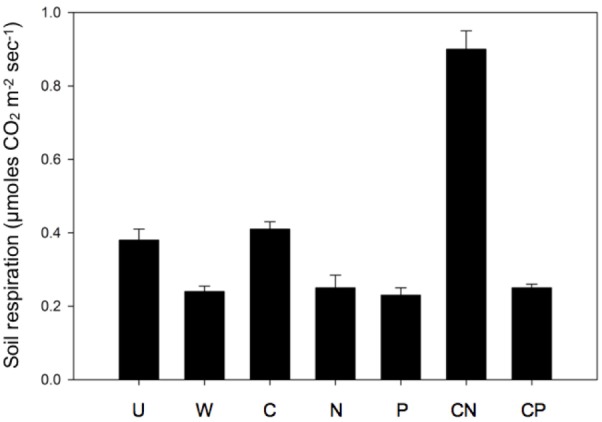
Soil respiration dramatically increased following CN additions. Values are means ± SEM (*n* = 8) with letters indicating differences (*P* < 0.05) based on a one-way ANOVA and Tukey’s HSD test.

Only the CN resource addition elevated soil respiration 1 week following the nutrient additions. As a result, we observed a 114–234% increase in soil respiration in CN soils compared to all other treatments (one-way ANOVA, *df* = 6, *F* = 11.6, *P* < 0.001, **Figure 5**). The soil treatments exhibited no differences in temperature at the time of sampling (one-way ANOVA, *df* = 6, *F* = 1.46, *P* = 0.24). The mean temperature of all treatments was 7.2°C ± 0.32 SD (*n* = 56).

## Discussion

Stoichiometric shifts of C:N:P in the coldest and driest soils on Earth (Virginia and Wall, 1999) alleviated resource limitations, created species-specific bacterial responses, and altered ecosystem processes. Our long-term coupled resource additions dramatically altered soil C:N:P leading to increases in inorganic N and P availability, but only a slight increase in soil organic C content, which was presumably consumed by bacteria. As hypothesized, C, N, CN, and CP additions created unique communities, relative to untreated soils, with CN and N having the most pronounced effect on bacterial species responses. We found that the alleviation of a C and N co-limitation facilitated the dominance of an *Arthrobacter species* (family, Micrococcaceae) that ultimately elevated soil respiration, and that shifts in C:N ratios may remove nutrient constraints on bacteria enhancing species richness and diversity. Contrary to our hypothesis, the addition of P, even to our relatively P-rich soils (Aislabie et al., 2006; Barrett et al., 2007), helped create unique communities for all single and coupled resource additions.

### Colimitation of CN Facilitates Species Dominance and Enhanced Respiration

The colimitation of organic C, N, and/or P are common in marine and freshwater systems where the abundance of photoautotrophs to organoheterotrophs is often influenced by two or more nutrients (Bouvy et al., 2004; Howarth and Marino, 2006; Cherif and Loreau, 2007; Saito et al., 2008). In soils, co-limitation exists but is harder to identify due to the high levels of bacterial diversity and the wide variety of resource substrates for species to exploit, from extremely labile C substrates to recalcitrant soil organic matter. Over the 6 years of the present study, as CN limitations were eased, (e.g., C:N decreased and N:P increased in CN amended soils), an *Arthrobacter* species (family = Micrococcaceae, Actinobacteria) went from being rare (0.06% ± 0.05) to dominant (47% ± 5.6). *Arthrobacter* species are common psychrotrophs found in Adelie penguin guano (Zdanowski et al., 2004) and Antarctic epilithic lichens (Selbmann et al., 2010) seeming to capitalize on the localized nutrient-rich penguin feces in the otherwise nutrient poor landscape. *Arthrobacter* strains can respond quickly to changes in nutrient conditions by breaking dormancy and growing within an hour of the removal of starvation stress (Soyanikova et al., 2017). Compared to temperate *Arthrobacter* species, Antarctic *Arthrobacter* possess lower metabolic versatility (Dsouza et al., 2015) but similar genes to many psychrophilic/psychrotolerant species (e.g., cold active hydrolytic enzymes; sigma factors; signal transduction pathways; carotenoid biosynthesis pathway; and genes induced by cold-shock, oxidative, and osmotic stresses (Dsouza et al., 2015; Singh et al., 2016). Further, our unclassified *Arthrobacter* OTU falls within a genus whose members readily decompose almost any algal and cyanobacterial bioproducts, from cyanotoxins (Lawton et al., 2011) to cellobiose, the final derivative of cellulose utilization (Schellenberger et al., 2011). Thus, the *Arthrobacter* we recovered is likely a well-adapted psychrophile poised to exploit common bacterial and algal derived C sources when N is available.

The functional consequences of *Arthrobacter* dominance were easily distinguishable. Often the functional consequences of soil bacterial community change is exceptionally difficult due to discern due to levels of functional redundancy (Yin et al., 2000; Miki et al., 2014) and large fractions of bacterial diversity being dormant or metabolically inactive at any given time (Lennon and Jones, 2011). In contrast, only in soils where *Arthrobacter* bacteria achieved dominance did soil respiration dramatically increase (114–234%). Our results are consistent with the findings of Hopkins et al. (2006) who showed that the addition of glucose, glycine, and ammonium stimulated the mineralization of lacustrine detritus and soil organic matter across different geomorphically defined landscapes in Garwood Valley, Antarctica. Even though the link between our dominant species and respiration is implied rather than explicit, dominant or abundant bacteria often contribute proportionally to universal soil processes such as respiration (Pedros-Alio, 2012). Thus, *Arthrobacter* most likely exploited mannitol and ammonium or nitrate to a greater extent than other species to become more metabolically active. Bacterial competition for essential resources may loosely be classified into two competition categories, scramblers and contesters (Hibbing et al., 2010). Scramble competition or exploitation competition involves rapid utilization of resources without directly interacting with other bacteria, while contest competition or interference competition involves direct antagonistic interactions between competitors. While both scramblers and contesters occur in most soils, the effects of competition for resources on bacterial taxa are often only implied (Zhou et al., 2002; Freilich et al., 2011) and potential interactions among limiting nutrients are often neglected (Fanin et al., 2015). In our CN-enriched soils, *Arthrobacter* is most likely a scrambler, better suited to capitalize on emerging resources. Its rise to dominance resulted in a decline in bacterial evenness and diversity while allowing for the persistence of rare taxa, as evidenced by similar species richness levels exhibited among the different resource treatments.

### Inorganic N Opened New Bacterial Niches for Rare Taxa

The removal of ammonium limitations opened new niches for once rare taxa to exploit. With the immense elevation of ammonium levels following N additions, bacterial richness increased upward of 48% in comparison to soils that receive only C or P additions. Higher levels of ammonium increased bacterial richness directly and indirectly by potentially stimulating nitrifying bacteria relying on ammonium and nitrite. We found that ammonium additions enhanced the abundance of two nitrite oxidizing *Nitrospira* species from the family Nitrosomonadaceae. Antarctic *Nitrospira* species may also contain amoA sequences (Magalhaes et al., 2014) and participate in complete nitrification (Daims et al., 2015). Our findings are consistent with the suggestion that the extreme abiotic severity of the McMurdo Dry Valley soil habitat drives the presence of ammonia oxidizing bacteria (AOB) like *Nitrospira* (Geyer et al., 2014; Magalhaes et al., 2014). Only after the soils became considerably less harsh with N additions were AOB found in soils. Presumably the increase in nitrite triggered certain species to increase in abundance. For example, six Gemmatimonadaceae species with the ability to reduce nitrite to nitric oxide via the NirK gene (clade II) (Decleyre et al., 2016) increased in abundance at least 1.8-fold under N additions relative to all other treatments. Also, four Rhodobacteraceae taxa with a high affinity for ammonium, characterized by high transcript abundance for ammonium transporters (Pfreundt et al., 2016), increased in abundance as N limitations were lifted. Many of these taxa responding to ammonium and possibly nitrite were rare (Sogin et al., 2006) with an abundance in untreated soils <0.1% of the total recovery (Aanderud et al., 2015). Thus, the enhanced availability of reduced forms of N following N-amendments stimulates microbial biodiversity in Antarctic soils.

### CP and P Deconstructed Species Assemblages

Lower soil C:P following P additions disrupted community co-occurrence patterns and facilitated new nutrient-related interactions among taxa. Even an increase in soil P, a nutrient that was not predicted to be limiting based on the initial soil C:N:P ratios, influenced species interactions. Excess soil P disrupted potential interactions among community assemblages, as evidenced by more than 50% of the network (i.e., significant species and interactions) disappearing and 14 taxa from three families (i.e., Phycisphaerae unclassified, Intrasporangiaceae, and Xanthomonadaceae) vanishing from the co-occurrence network. However, as P limitations were alleviated a more diffuse and less connected network potentially centered on P availability emerged. For example, two new Trueperaceae species (*Deinococcus*) were incorporated into the model and the collective abundance of the three Trueperaceae species was 13-times higher in P amended soils. Members of the Trueperaceae family are remarkably resistant to ionizing radiation and able to grow under multiple extreme conditions, including alkaline, moderately saline, and high temperature environments (Ivanova et al., 2011). Deinococcus-Thermus taxa in general possess a remarkable number of genes encoding for catabolic enzymes including phosphatases (Daly et al., 2010), suggesting that the access to P is potentially linked to radiation resistance and/or helps boost the survival of Trueperaceae in extreme soils. Additionally, multiple shared taxa between our two networks [i.e., Spartobacteria unclassified (Verrucomicrobia), Micrococcaceae (Actinobacteria), and Chitinophagaceae (Bacteroidetes)] increased in abundance under P additions. The abundance of taxa within these families may track the availability of P in soils and water (Johnson et al., 2017; Wang et al., 2017). Even with P additions communities seemed to remain partially reliant on Solirubrobacteriaceae taxa. Members of the Solirubrobacteriaceae family may enhance the weathering of volcanic rocks, which are common in soils occurring on Ross Sea Till (Cockell et al., 2013). Despite P additions, Solirubrobacteriaceae taxa were integral to all models constituting upward of 10% of species and 20% of the recovery suggesting that mineral weathering is essential to enhance micronutrient availability under both high and low nutrient conditions. As soils transition from a nutrient-poor to a nutrient-rich state, the excess soil P potentially disrupted interactions among bacterial taxa. For example, the addition of water and organic matter in McMurdo Dry Valley soils caused certain bacteria (members of the and Actinobacteria, Proteobacteria, and Firmicutes) to become active demonstrating a potential taxonomical shift from species adapted to dry oligotrophic to moist copiotrophic conditions (Buelow et al., 2016). Thus, future climate-driven changes that ameliorate the current stoichiometric imbalances of the dry valley soils (Nielsen and Ball, 2014; Gooseff et al., 2017), may deconstruct current bacterial communities and reorganizing them into communities dominated by more copiotrophic taxa. Co-occurrence networks do provide insights into potential interactions among taxa within a community (Freilich et al., 2011; Freedman and Zak, 2015), but to fully understand interactions among bacteria a more direct approach is needed.

### Annual Water Additions Failed to Elicit Bacterial Response

Water is necessary for imbalances in stoichiometric nutrient ratios to influence communities; however, our one-time water addition alone was not enough to create lasting effects on bacteria community structure. Frequent water additions do influence bacterial activity across Dry Valleys (Schwartz et al., 2014; Buelow et al., 2016) where soil moisture is ephemeral and extremely patchy. Surface hydrogeological features such as water tracks in soils (Levy et al., 2014), lateral margins of stream and lake margins (Zeglin et al., 2011), and discontinuous patches of soils often form in the same location due to wind sheltering and microtopography (Gooseff et al., 2003) enhance bacterial metabolic activity and alter species distributions.

## Conclusion

Stoichiometric additions of C, N, and P reduced resource limitations, created species-specific bacterial responses, and in one case altered a fundamental ecosystem process. The most dramatic effects of changes in ecosystem stoichiometry occurring around C and N additions in our initially N limited soils. C as mannitol and N as equal molar concentrations ammonium and nitrate induced an almost twofold reduction in C:N ratios; caused bacterial evenness and diversity to decline; allowed one rare Micrococcaceae, an *Arthrobacter* species, to dominate community abundance; and elevated soil respiration by 136% compared to untreated soils. N additions alone also reduced C:N ratios, and in contrast to CN-additions, increased species richness and diversity by at least 48 and 13%, respectively, compared to soils receiving a single resource as C or P, and enhanced the abundance of rare taxa dependent on N for metabolism and growth. The addition of P to levels well below the C:P ratio necessary for balanced microbial growth also influenced soil microbial communities. Based on community co-occurrence networks, lower C:P ratios in soils following P and CP additions reduced the number of taxa interacting with one another by 51% and the number of interactions among taxa by 73% relative to untreated and watered soils. Our results suggest that the alleviation of C and N co-limitation facilitated the dominance of single species ultimately altering ecosystem processes; the reduced forms of inorganic N open multiple niches for bacteria to exploit, and excess soil P disrupted interactions within communities.

## Author Contributions

ZA, SS, BB, DW, JB, RV, and BA conducted the experiments. ZA, SS, BB, DW, JB, MM, NG, RV, AB, and BA analyzed and interpreted the data. ZA, SS, DW, JB, NG, MM, RV, AB, and BA helped to write and review the manuscript. ZA agrees to be accountable for all aspects of the work in ensuring that questions related to the accuracy or integrity of any part of the work are appropriately investigated and resolved.

## Conflict of Interest Statement

The authors declare that the research was conducted in the absence of any commercial or financial relationships that could be construed as a potential conflict of interest.

## Footnotes

1 http://dnac.byu.edu/

2 https://www.mothur.org/wiki/454_SOP
