# Corrigendum: Stoichiometric Shifts in Soil C:N:P Promote Bacterial Taxa Dominance, Maintain Biodiversity, and Deconstruct Community Assemblages

**DOI:** 10.3389/fmicb.2019.00391

**Published:** 2019-03-12

**Authors:** Zachary T. Aanderud, Sabrina Saurey, Becky A. Ball, Diana H. Wall, John E. Barrett, Mario E. Muscarella, Natasha A. Griffin, Ross A. Virginia, Albert Barberán, Byron J. Adams

**Affiliations:** ^1^Department of Plant and Wildlife Sciences, Brigham Young University, Provo, UT, United States; ^2^School of Mathematical and Natural Sciences, Arizona State University, Phoenix, AZ, United States; ^3^Department of Biology, School of Global Environmental Sustainability, Colorado State University, Fort Collins, CO, United States; ^4^Department of Biological Sciences, Virginia Polytechnic Institute, Blacksburg, VA, United States; ^5^Department of Plant Biology, University of Illinois Urbana-Champaign, Champaign, IL, United States; ^6^Environmental Studies Program, Dartmouth College, Hanover, NH, United States; ^7^Department of Soil, Water and Environmental Science, University of Arizona, Tucson, AZ, United States; ^8^Evolutionary Ecology Laboratories, and Monte L. Bean Museum, Department of Biology, Brigham Young University, Provo, UT, United States

**Keywords:** ecological stoichiometry, Lake Fryxell Basin, McMurdo Dry Valleys, network community modeling, nutrient colimitation, Solirubrobacteriaceae

“Albert Barberán” was not included as an author in the published article. The correct Author Contributions statement appears below:

“ZA, SS, BB, DW, JB, RV, and BA conducted the experiments. ZA, SS, BB, DW, JB, MM, NG, RV, AB, and BA analyzed and interpreted the data. ZA, SS, DW, JB, NG, MM, RV, AB, and BA helped to write and review the manuscript. ZA agrees to be accountable for all aspects of the work in ensuring that questions related to the accuracy or integrity of any part of the work are appropriately investigated and resolved.”

The authors apologize for this error and state that this does not change the scientific conclusions of the article in any way. The original article has been updated.

