# Supplementary material for: Stoichiometric Shifts in Soil C:N:P Promote Bacterial Taxa Dominance, Maintain Biodiversity, and Deconstruct Community Assemblages
Source: Front Microbiol. 2018 Jul 3;9:1401. doi: 10.3389/fmicb.2018.01401 (PMC6037766; doi:10.3389/fmicb.2018.01401)
Supplement: Supplementary file 1 [file Data_Sheet_1.docx]

**SUPPLEMENTAL FIGURE 1 |** Distribution of nine phyla and three subclasses in resource additions soils. Treatments abbreviations are outlined in supplemental figure 1. Values are the relative recovery of major taxonomical groups (recovery ≥ 1.0%) in three samples for each treatment based on rDNA libraries.

**
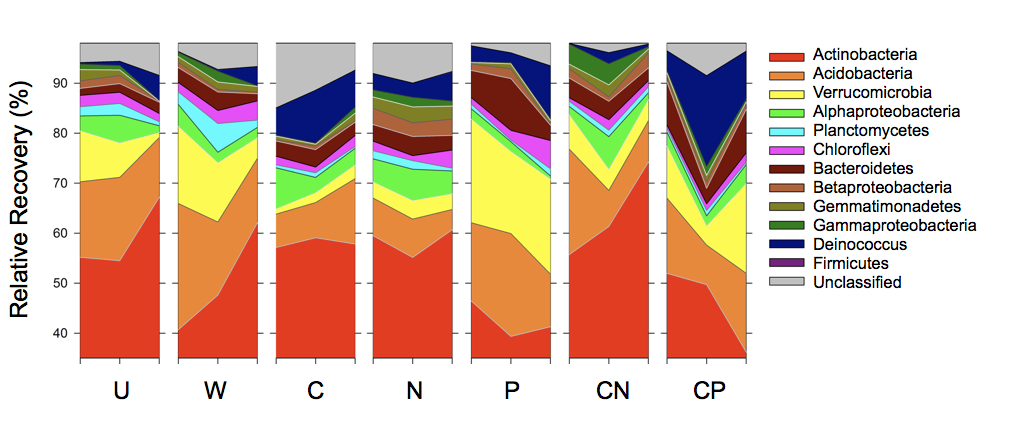
**
